# Differences in gut microbiota and faecal metabolomics characteristics in preterm infants with feeding intolerance

**DOI:** 10.1099/jmm.0.002138

**Published:** 2026-03-26

**Authors:** Jinya Wang, Jiejing Zhang, Ji Wang, Danqiong Lu, Shiwei Lai, Xinyu Wang

**Affiliations:** 1 NICU, Zhoushan Women and Children Hospital, Zhoushan 316000, PR China

**Keywords:** feeding intolerance, gut microbiota, metabolomics, preterm infants

## Abstract

**Introduction.** Feeding intolerance (FI) is one of the most common clinical issues in preterm infants, and there are currently no internationally unified diagnostic criteria.

**Gap Statement.** Screening valuable biomarkers and evaluating their diagnostic value for FI in preterm infants is of great significance.

**Aim.** This study aimed to identify and assess diagnostic biomarkers for feeding intolerance in preterm infants.

**Methodology.** This study included clinical data from 49 preterm infants admitted to a tertiary maternal and child health hospital in Zhejiang’s coastal region (January to June 2024). Based on feeding assessments at day 21 postpartum recorded in electronic medical records, infants were divided into feeding-tolerant (FT, *n*=34) and feeding-intolerant (FI, *n*=15) groups. Patient data analysis incorporated maternal age, gestational age, parity, antibiotic use, pregnancy complications and neonatal factors (birth weight, Apgar scores, delivery/feeding methods, vomiting, abdominal distension, gastric residuals, kangaroo care and enema use). Faecal samples underwent microbiome and metabolomic profiling to identify diagnostic biomarkers.

**Results.** Baseline data showed no significant differences in maternal-infant characteristics between groups (*P>*0.05). Dynamic monitoring of feeding tolerance in 21-day-old preterm infants revealed that the incidence of vomiting, abdominal distension, abnormal intestinal morphology and gastric residual volume >30% or >2 ml kg^−1^ was significantly higher in the FI group than in the FT group (*P*<0.001), whereas there was no significant difference in the frequency of nasogastric feeding between the two groups (*P*>0.05). Microbial analysis revealed enrichment of *Escherichia* (10.92%) and *Klebsiella* (6.88%) in FT infants, while FI infants specifically harboured increased *Clostridium_P* (3.93%), *Burkholderia* (4.06%) and *Limosilactobacillus* (4.94%). Metabolomic profiling identified significant pathway differences in ATP-binding cassette transporters (ABC transporters), carbohydrate digestion/absorption and propanoate metabolism. The receiver operating characteristic (ROC) analyses showed that metabolites arginine–proline (Arg–Pro, AUC=0.920), glutamic acid–arginine (Glu–Arg, AUC=0.873), lactaldehyde (AUC=0.900) and genera *Clostridium*_*P* (AUC=0.947), *Escherichia* (AUC=0.765), *Staphylococcus* (AUC=0.733) and *Bifidobacterium* (AUC=0.851) exhibited robust predictive value for FI.

**Conclusion.** Our study demonstrates that bacterial genera such as *Staphylococcus*, *Clostridium*_*P*, *Bifidobacterium* and *Escherichia* in the gut microbiota, along with metabolites including Arg–Pro, Glu–Arg and lactaldehyde identified in metabolomics, can serve as diagnostic criteria for feeding tolerance in preterm infants. *Klebsiella* shows a certain degree of diagnostic efficacy but falls into the category of ‘low accuracy’, requiring comprehensive evaluation considering the research background, sample characteristics and clinical context.

## Data Summary

The raw 16S rRNA gene sequencing data generated in this study have been deposited in the National Center for Biotechnology Information (NCBI) Sequence Read Archive under BioProject accession number PRJNA1406357. The individual sample accession numbers can be found in Table S1, available in the online Supplementary Material.

## Introduction

Preterm infants are live-born neonates with a gestational age of less than 37 weeks. Their organ development and adaptive capacity are poorer than those of full-term neonates, resulting in a relatively lower survival rate [1]. Feeding intolerance (FI) is currently one of the most common clinical problems in preterm infants, mainly caused by insufficient digestive system development and gastrointestinal dysfunction [2]. Common symptoms include abdominal distension, frequent vomiting and feeding difficulties, which prevent adequate nutrient intake and severely impair growth and development. These issues may also trigger serious complications such as necrotizing enterocolitis, threatening infant survival [3,4]. Epidemiological data show that the incidence of FI in preterm infants reaches 53.45%, and in very low birth weight infants, it reaches 48.57% [4]. However, the current diagnosis of FI in preterm infants mainly relies on subjective judgement criteria represented by indicators, such as gastric residual volume, abdominal distension, vomiting or feeding outcomes, and there is a lack of unified objective diagnostic criteria. Therefore, it is necessary to screen more specific biomarkers as indicators to provide an objective diagnostic basis for the diagnosis of FI in preterm infants.

The gut microbiota is considered the host’s ‘second genome’. It plays a key role in nutritional metabolism, immune regulation and maintaining the intestinal barrier. Studies have shown that the imbalance of gut microbiota in preterm infants (such as the decrease in the abundance of beneficial bacteria and the enrichment of conditional pathogens) is closely associated with the occurrence of feeding intolerance [5]. Short-chain fatty acids (SCFAs) mediate communication between the gut microbiota and the immune system. They are closely linked to gastrointestinal motility, mucosal barrier structure and digestive–absorptive functions [6]. Most SCFAs, such as acetic acid, propionic acid and butyric acid, exist predominantly in ionic form within the body. Excessive accumulation of SCFAs can lead to FI and, in severe cases, trigger necrotizing enterocolitis (NEC) [7]. Studies have shown that faecal butyric acid levels in NEC infants are significantly lower than those in healthy infants [8]. In addition, faecal metabolomics can capture the changes in metabolites produced by the interaction between intestinal micro-organisms and the host [9], providing direct evidence for disease mechanism research and demonstrating unique advantages in making up for the diagnostic defects of FI. Furthermore, combined analysis of gut microbiota and metabolomics creates a complete evidence chain linking ‘microbe, metabolite and phenotype’. An imbalance in microbiota structure affects intestinal function by altering metabolite profiles. Meanwhile, metabolomic data offer direct functional validation for microbiota research. However, existing studies have mostly focused on changes in a single type of flora or metabolite, lacking a systematic analysis of the overall ‘flora-metabolite-disease phenotype’ network. This makes it difficult to comprehensively reveal the mechanism underlying the occurrence and development of feeding intolerance, which also limits the screening of potential diagnostic biomarkers. Therefore, from the perspective of multi-omics integration, combining the structural characteristics of gut microbiota with changes in metabolite profiles to deeply explore their synergistic role in feeding intolerance in preterm infants is of great significance for the accurate identification of diagnostic biomarkers.

This study explores the interaction mechanisms between the gut microbiota and the host. We use quantitative analysis of microbial metabolites as a key bridge to connect the microbial community with clinical phenotypes. Specifically, by detecting and analysing metabolites in the faeces of preterm infants, we aim to evaluate the diagnostic value of SCFAs for FI in this population. We seek to identify microbiota and metabolomic biomarkers with diagnostic value. The ultimate goal is to provide an objective reference standard for the diagnosis of FI in preterm infants based on these biomarkers.

## Methods

### Participants and groups

**Fig. 1.**
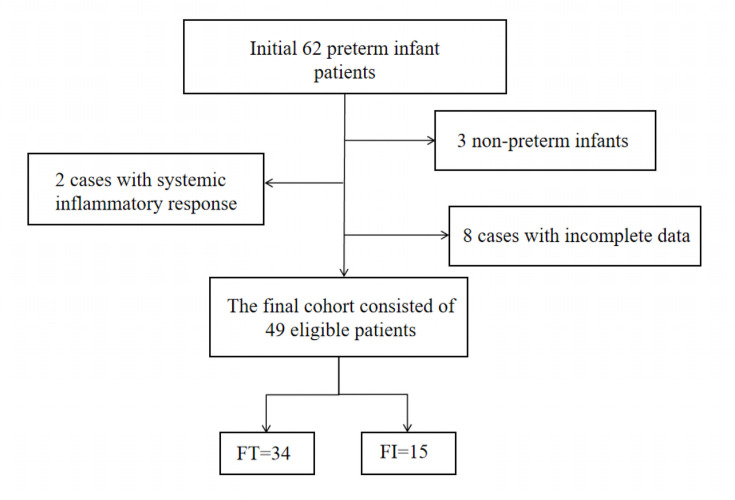
Study flow diagram.

This retrospective cohort study was conducted at a tertiary Class A maternal and child health hospital in Zhejiang Province, China. Based on a priori power analysis (*α*=0.05, *β*=0.20, p1=0.27, p2=0.73) using PASS 15.0 software, the minimum required sample size was determined to be 30 cases. From January to June 2024, we initially screened 62 preterm infants. We excluded 13 infants due to severe diarrhoea (*n*=3), systemic inflammatory response (*n*=2) and incomplete data (*n*=8), resulting in a final cohort of 49 eligible infants. These infants were stratified into feeding-tolerant (FT group, *n*=34) and feeding-intolerant (FI group, *n*=15) groups based on a 21-day feeding evaluation from the EMR system (Fig. 1). The study protocol was approved by the Institutional Review Board/Ethics Committee (approval no: KY2022009) in accordance with national regulations and the Declaration of Helsinki, and all data were de-identified to protect patient confidentiality.

### Case definition

Diagnostic criteria: According to the diagnostic criteria for FI recommended by the *Clinical Guidelines for Diagnosis and Treatment of Feeding Intolerance in Preterm Infants (2020)* [10], FI was defined as meeting at least one of the following criteria: (1) gastric residual volume (GRV) ≥50% of the previous feeding volume every 3 h (q3h); (2) frequent vomiting (≥3 episodes per day) and/or abdominal distension; or (3) disruption of enteral feeding plans (including reduction, delay or cessation of feeding). Infants who did not meet any of these criteria at 21 days of age were assigned to the feeding-tolerant (FT) group, while those who met any one criterion were assigned to the FI group.

Inclusion criteria: (1) approval by the hospital ethics committee with written informed consent obtained from legal guardians; (2) gestational age less than 37 weeks; (3) exclusive breastfeeding or formula feeding for more than 5 days before specimen collection; (4) absence of meconium-stained amniotic fluid or premature rupture of membranes at birth; (5) no congenital diseases affecting intestinal function; (6) meeting diagnostic criteria; and (7) complete clinical records including paired stool and peripheral blood samples.

Exclusion criteria: (1) uncooperative guardians; (2) infants with systemic inflammatory response syndrome, Crohn’s disease, inflammatory bowel disease, congenital gastrointestinal anomalies, NEC or severe diarrhoea during hospitalization; (3) severe birth asphyxia, coagulation disorders or those undergoing abdominal surgery; (4) use of intestinal probiotics; and (5) antibiotic use within the past week.

### Data collection

#### Data collection and quality control

This study utilized data from the hospital’s electronic medical record (EMR) system, laboratory database and biobank. Clinical data were collected from preterm infants at 21 days of age. These data included maternal age, obstetric history, delivery mode, antenatal antibiotic exposure, neonatal sex, gestational age, birth weight, Apgar scores at 1 and 5 min, feeding regimen (initiation time and type), vomiting episodes, abdominal distension, gastric residual volume, kangaroo mother care and enema administration. All clinical data were extracted from the EMR. Faecal specimens preserved at −80°C for gut microbiota and metabolomic analyses were retrieved from the biobank. To ensure data integrity, we implemented a standardized extraction protocol. Two independent researchers cross-verified all records, with discrepancies resolved by a senior clinician. Extreme values were cross-checked against original laboratory reports to exclude technical errors.

#### Specimen collection

Faecal samples were aseptically collected from spontaneously passed stools of preterm infants (5–7 days postnatal age) using sterile spoons. Approximately 2 g of the mid-portion faeces was transferred to sterile containers, immediately flash-frozen at −20 °C, transported on dry ice and subsequently stored at −80 °C.

#### Gut microbiota analysis

After taking the materials out of the freezer, aliquots weighing between 0.2 and 0.5 g were quickly weighed and put into centrifuge tubes filled with extraction lysis buffer so they could be ground. A homogenizer with a frequency of 60 Hz was used for the grinding process. The OMEGA Soil DNA Kit (D5635-02, Omega Bio-Tek, Norcross, GA, USA) was then used to extract DNA from the processed samples in accordance with the manufacturer’s instructions. The extracted DNA was measured using a NanoDrop spectrophotometer and assessed by 0.8% agarose gel electrophoresis to determine the fragment size distribution. The V3–V4 hypervariable regions of bacterial 16S rRNA genes were amplified with PCR primers and subjected to paired-end sequencing (PE300) on an Illumina MiSeq platform.

Microbiome bioinformatics analysis was performed using QIIME 2 (v2019.10) and included the following: (1) alpha diversity analysis (e.g. Chao1 index) to evaluate microbial richness; (2) beta diversity analysis to compare inter-group structural differences in bacterial communities; (3) taxonomic composition analysis at multiple classification levels; and (4) differential species analysis to identify significant variations in microbial community structure between groups.

#### Metabolomics analysis

Precisely weighed 25 mg aliquots of frozen samples were homogenized in EP tubes, and tissue metabolites were extracted following the manufacturer’s protocol.

Metabolite extraction: The samples (25–1 mg) were taken and mixed with beads and 500 uµl of extraction solution (MeOH/ACN/H2O, 2 : 2 : 1 (v/v)). The extraction solution contains deuterated internal standards. The mixed solution was vortexed for 30 s. Then, the mixed samples were homogenized (35 Hz, 4 min) and sonicated for 5 min in a 4 °C water bath, and the step was repeated three times. The samples were incubated for 1 h at −40 °C to precipitate proteins. Then, the samples were centrifuged at 12,000 r.p.m. for 15 min at 4 °C. The supernatant was transferred to a fresh glass vial for analysis. The quality control (QC) sample was prepared by mixing an equal aliquot of the supernatant of the samples.

LC-MS/MS analysis: For polar metabolites, LC-MS/MS analyses were performed using a UHPLC system (Vanquish, Thermo Fisher Scientific) with a Waters ACQUITY UPLC BEH Amide (2.1 mm; 50 mm, 1.7 um) coupled to an Orbitrap Exploris 120 mass spectrometer (Orbitrap MS, Thermo). The mobile phase consisted of 25 mmol l^−1^ ammonium acetate and 25 ammonia hydroxide in water (pH=9.75) (A) and acetonitrile (B). The auto-sampler temperature was 4 °C, and the injection volume was 2 µl. The Orbitrap Exploris 120 mass spectrometer was used for its ability to acquire MS/MS spectra on information-dependent acquisition mode in the control of the acquisition software (Xcalibur, Thermo). In this mode, the acquisition software continuously evaluates the full-scan MS spectrum. The ESI source conditions were set as follows: sheath gas flow rate as 50 Arb, Aux gas flow rate as 15 Arb, capillary temperature 320 °C, full MS resolution as 60,000, MS/MS resolution as 15,000, collision energy: SNCE 20/30/40, spray voltage as 3.8 kV (positive) or −3.4 kV (negative), respectively.

Multidimensional statistical analyses identified significantly differentiated metabolites, including (1) abundance profiling, such as sample correlation analysis and hierarchical clustering heatmaps, and (2) pairwise comparative analyses, including univariate statistics, principal component analysis (PCA), partial least squares discriminant analysis, orthogonal projections to latent structures discriminant analysis (OPLS-DA), differential metabolite clustering, correlation analysis of differential compounds and (KEGG) Kyoto Encyclopedia of Genes and Genomes pathway enrichment.

### Statistical analysis

Taxonomic assignment of ASVs was conducted using QIIME2’s Naive Bayes classifier (v2019.10) against the silva 132 99% OTU (Operational Taxonomic Unit) reference database [11,12]. All graphic drawing is done using the R package (v4.5.2). Venn diagrams were generated to visualize shared and unique features among samples, while KRONA (v2.7.1) was employed for interactive taxonomic visualization [13]. Alpha diversity was assessed using richness estimators (Chao1, observed species, Good’s coverage and Fisher’s alpha) and evenness metrics (Shannon, Simpson and ENSPIE). Linear discriminant analysis effect size was performed to identify differentially abundant biomarkers among groups with effect size estimation [14]. ProteoWizard (v3.0.6428) was used to convert the raw metabolomics data to.mzML format. XCMS (a chromatographic data processing program, online v3.7.1) was then used to process the data for peak alignment, retention time correction and peak area extraction. For XCMS parameter configuration: (1) the centWave method was used to find peaks with a 10 p.p.m. m/z tolerance, chromatographic peak width range of 10–60 s and prefilter thresholds (minimum 10 peaks per scan with intensity ≥100) and (2) peak grouping parameters included a 5-s retention time window bandwidth (bw), 0.025 Da m/z deviation tolerance (mzwid) and minimum detection fraction of 0.5. A one-way ANOVA was conducted using SPSS 26.0 to identify significant differences between groups (*P*<0.05) when the assumptions of normality and homogeneity of variance were satisfied. In order to generate the ROC curves, compare area under the curve (AUC) values and compute sensitivity/specificity for assessing diagnostic efficacy, the screening findings from metabolomics and species diversity analyses were utilized as dependent variables.

## Results

### Clinical characteristics of enrolled subjects

This study involved 49 preterm infants, of whom 34 were in the FT group and 15 were in the FI group (Table 1). Among the two groups of preterm infants, baseline data revealed no statistically significant variations in sex, Apgar scores, frequency of kangaroo care, feeding technique, gestational age, birth weight or mode of delivery (*P*>0.05). Maternal factors, including age, parity, number of pregnancies, rate of antibiotic use during pregnancy and the frequency of pregnancy problems, did not differ statistically across groups (*P*>0.05). These results revealed that the two groups of patients had good comparability.

**Table 1.** Clinical characteristics of enrolled subjects

Variable	FT (*n*=34)	FI (*n*=15)	*Z/t/X* ^*2*^	*P*-value
Maternal age (years, $\overset{-}{x}\pm s$)	32.06±4.80	33.87±3.85	−1.284	0.205
Gravidity [number, *M*(*P_25_*, *P_75_*)]	2.15 (1.00, 3.00)	2.6 (1.00, 4.00)	−1.399	0.162
Parity [number, *M*(*P_25_*, *P_75_*)]	1.19 (1.00, 1.00)	1.45 (1.00, 2.00)	−1.679	0.093
Pregnancy complications (*n*, %)			2.118	0.146
Yes	19 (55.90)	5 (33.30)		
No	15 (44.10)	10 (66.70)		
Gestational age (weeks, $\overset{-}{x}\pm s$)	33.21±1.67	32.88±1.85	0.619	0.539
Birth weight (g, $\overset{-}{x}\pm s$)	1,972.35±391.14	1,766.00±435.71	1.644	0.107
Feeding methods (*n*, %)			0.567	0.451
Exclusive breastfeeding	12 (35.30)	7 (46.70)		
Formula feeding	22 (64.70)	8 (53.30)		
Apgar [1 min, *M*(*P_25_*, *P_75_*)]	8.91 (9.00, 9.00)	8.80 (8.00, 9.00)	−1.349	0.177
Apgar [5 min, *M*(*P_25_*, *P_75_*)]	9.79 (10.00, 10.00)	9.67 (9.00, 10.00)	−1.122	0.262
Antenatal antibiotics (*n*, %)			0.000	1.000
Yes	1 (2.90)	1 (6.70)		
No	33 (97.10)	14 (93.30)		
Mode of delivery (*n*, %)			0.004	0.951
Caesarean	23 (67.60)	11 (73.30)		
Vaginal	11 (32.40)	4 (26.70)		
Sex of preterm infant (*n*, %)			1.402	0.236
Male	22 (64.70)	7 (46.70)		
Female	12 (35.30)	8 (53.30)		
Kangaroo mother care (*n*, %)			0.164	0.686
Yes	16 (47.10)	8 (53.30)		
No	18 (52.90)	7 (46.70)		

### Results of feeding tolerance in preterm infants

This study dynamically monitored feeding tolerance in preterm infants at 21 days of age. Intestinal pattern alterations, vomiting, abdominal distension and gastric residual volumes above 30% or 2 ml kg^−1^ were all observed to be considerably more common in the FI group than in the FT group (*P*<0.001). The frequency of nasogastric feeding did not, however, differ significantly between the FI and FT groups (*P*>0.05) (Table 2).

**Table 2.** Feeding tolerance in preterm infants

Clinical symptom	FT (*n*=34)	FI (*n*=15)	*X* ^*2*^	*P*-value
Vomiting (*n*, %)			11.999	<0.001
No	34 (100.00)	9 (60.00)		
Yes	0 (0.00)	6 (40.00)		
Abdominal distension (*n*, %)			40.282	<0.001
No	33 (97.10)	0 (0.00)		
Yes	1 (2.90)	15 (100.00)		
Intestinal pattern (*n*, %)			31.825	<0.001
No	34 (100.00)	3 (20.00)		
Yes	0 (0.00)	12 (80.00)		
GRV >30% or > 2 ml kg^−1^ (*n*, %)			12.103	<0.001
No	31 (91.20)	6 (40.00)		
Yes	3 (8.80)	9 (60.00)		
NG feeding (*n*, %)			3.378	0.066
No	21 (61.80)	5 (33.30)		
Yes	13 (38.20)	10 (66.70)		

### Gut microbiota profiling

#### Gut microbial alpha diversity and beta diversity indices

**Fig. 2.**
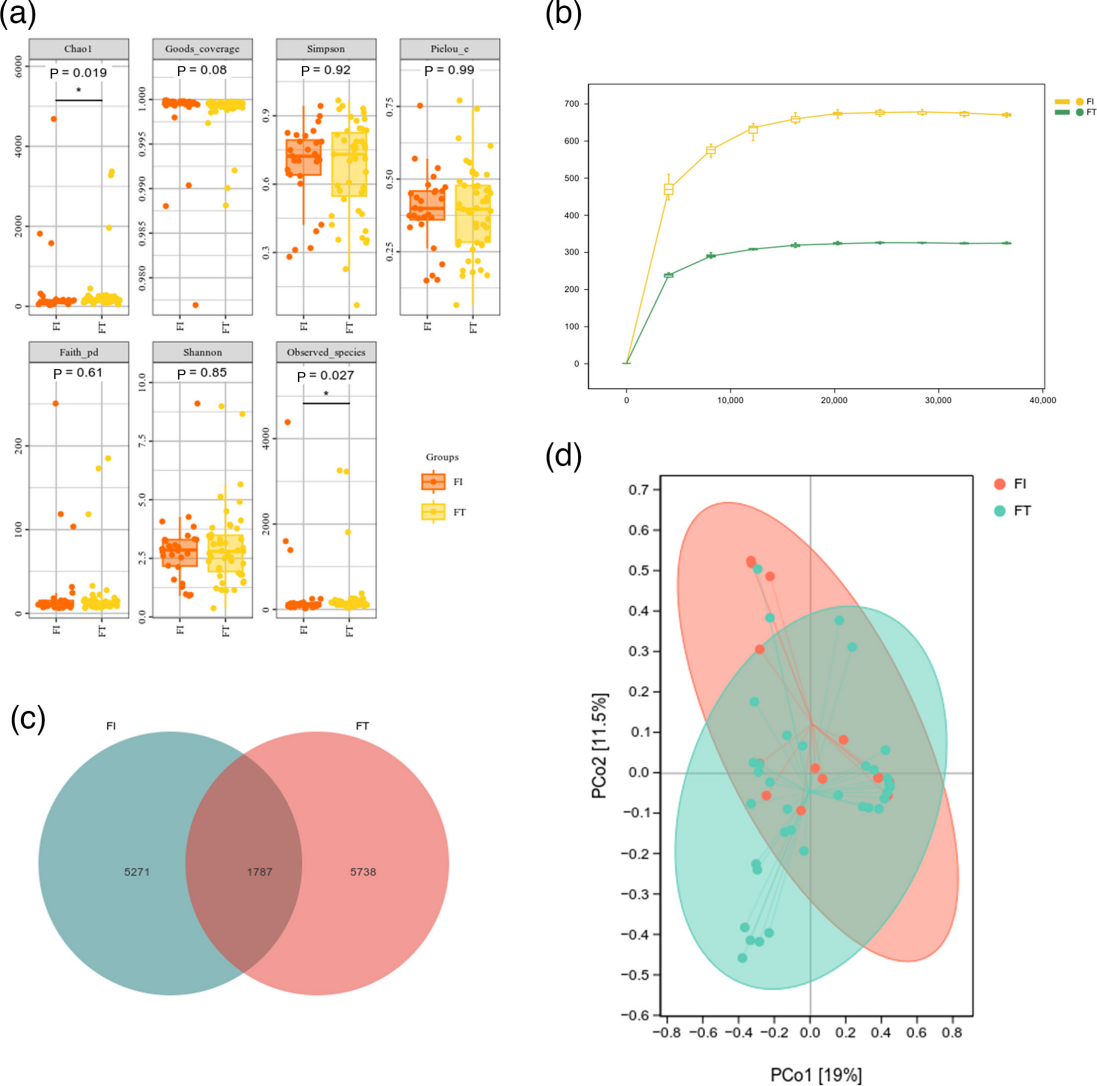
Gut microbiota diversity analysis. (**a**) Alpha diversity index. (**b**) Rarefaction curve. (**c**) The Venn diagram of the OTUs of each group. (**d**) Distance matrix and PCA.

Alpha diversity analysis showed statistically significant differences between the FT and FI groups in the Chao1 index (*P*=0.019) and Observed_species index (*P*=0.027), with both *P*-values <0.05. This indicates that preterm infants in the FI and FT groups exhibited distinct gut microbial diversity patterns. In contrast, no significant intergroup differences were observed for other diversity metrics, including Goods_coverage, Simpson, Pielou_e, Faith_pd and Shannon indices (*P*>0.05) (Fig. 2a). Each sample’s rarefaction curve was nearly saturated, there was a respectable quantity of sequencing data and there was enough sequencing depth (Fig. 2b). The gut microbiota of the two groups exhibited similar *β*-diversity features in key dimensions, as indicated by the partial overlap between the FI and FT groups in the principal coordinate analysis plot based on the distance matrix. However, an obvious clustering trend was observed on the principal coordinates: FI samples (in red) and FT samples (in cyan) showed partial separation in the ordination space defined by PCo1 (19% of variance explained) and PCo2 (15% of variance explained) (Fig. 2d). Twelve thousand seven hundred ninety-six representative OTUs were used to annotate the gut microbiota. Additionally, 1,787 OTUs were shared by the FI and FT groups, while 5,271 and 5,738 OTUs were found in each group, respectively (Fig. 2c).

#### Analysis of microbial community composition

**Fig. 3.**
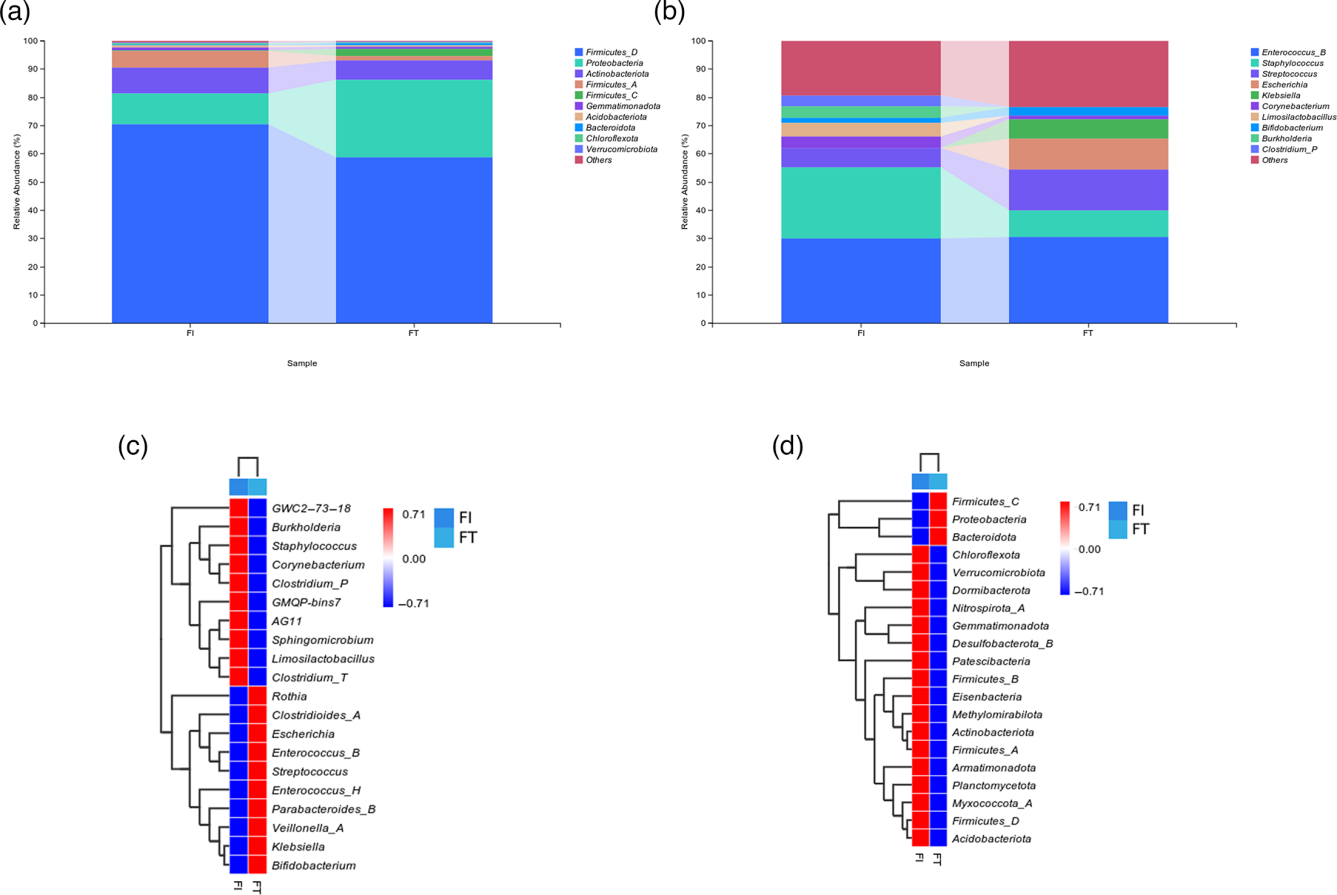
Composition and differential analysis of gut microbiota in preterm infants. (**a**) Analysis of species composition of the two groups of microbial communities at the phylum level. (**b**) Analysis of species composition of the two groups of microbial communities at the genus level. (**c**) Heatmap of species composition of the two groups of the phylum. (**d**) Heatmap of species composition of the two groups of the genus.

This study elucidates the structure of the microbial communities in the samples across different taxonomic levels. The gut microbiota of preterm newborns in both groups was primarily made up of phyla, such as *Firmicutes_D*, *Proteobacteria* and *Actinobacteriota*, according to the results (Fig. 3a). At the phylum level, there were no discernible or regular variations between the FI and FT groups in the relative abundance of gut flora. This suggests that both groups’ gut flora compositions were generally comparable at the phylum level in preterm newborns. Therefore, we further conducted an analysis at the genus level and found that the genera *Escherichia* and *Klebsiella* had relatively high proportions in the FT group (10.92%, 6.88%), while their proportions were 0% in the FI group. In contrast, the genera *Clostridium_P*, *Burkholderia* and *Limosilactobacillus* showed the opposite pattern. These three genera accounted for 3.93%, 4.06% and 4.94% respectively, in the FI group, but were absent (0%) in the FT group. Taken together, these findings suggest that there were significant differences in the composition of gut microbiota at the genus level between the two groups of preterm infants (Fig. 3b).

#### Heatmap analysis of differential gut microbiota

At the phylum level, various phyla showed different relative abundances between the two groups. Colour intensity indicated their association with the groups, with some phyla showing higher relative abundance in either the FI or FT group (Fig. 3c). Further analysis of the top 20 differential genera between the 2 groups revealed that their relative abundances exhibited inverse trends in the FI and FT groups. For example, genera such as *Burkholderia*, *Staphylococcus*, *Corynebacterium*, *Clostridium_P*, *Sphingomicrobium*, *Limosilactobacillus* and *Clostridium_T* showed significantly decreased expression abundances in the FT group, while their expression abundances were significantly increased in the FI group. In contrast, genera like *Rothia*, *Clostridioides_A*, *Escherichia*, *Enterococcus_B*, *Streptococcus*, *Enterococcus_H*, *Parabacteroides_B*, *Veillonella_A*, *Klebsiella* and *Bifidobacterium* showed significantly decreased expression abundances in the FI group, but significantly increased expression abundances in the FT group (Fig. 3d).

#### Association between gut microbiota alterations and feeding intolerance in preterm infants

Logistic regression on the relative abundances of 13 genera identified above revealed that only *Escherichia*, *Klebsiella*, *Clostridium*_*P*, *Bifidobacterium* and *Staphylococcus* were significantly associated with feeding intolerance in low birth weight preterm infants (*P*<0.05) (Table 3).

**Table 3.** Association between intestinal microbiota alterations and feeding intolerance in low birth weight preterm infants: a logistic regression analysis

Genus	B	se	Wald	*P*-value	OR	95% CI
*Enterococcus_B*	−0.055	0.439	0.016	0.901	0.947	0.401–2.237
*Streptococcus*	−0.634	0.618	1.051	0.305	0.530	0.158–1.783
*Bifidobacterium*	1.905	0.844	5.100	0.024	6.719	0.735–0.967
*Escherichia*	−6.371	2.363	7.272	0.007	0.002	0.00–0.175
*Klebsiella*	−3.572	1.492	5.731	0.017	0.028	0.002–0.523
*Clostridium_P*	12.054	3.593	11.253	<0.001	171.306	150.098–196.612
*Staphylococcus*	1.083	0.497	4.748	0.029	2.953	1.115–7.820
*Corynebacterium*	0.905	0.875	1.071	0.301	2.473	0.445–13.739
*Sphingomicrobium*	1.339	1.951	0.471	0.493	3.816	0.083–174.885
*Veillonella_A*	−0.827	1.413	0.343	0.558	0.437	0.027–6.970
*Rothia*	0.830	0.962	0.744	0.388	2.293	0.348–15.107

#### Analysis of quality control and expression abundance of metabolites in two groups

**Fig. 4.**
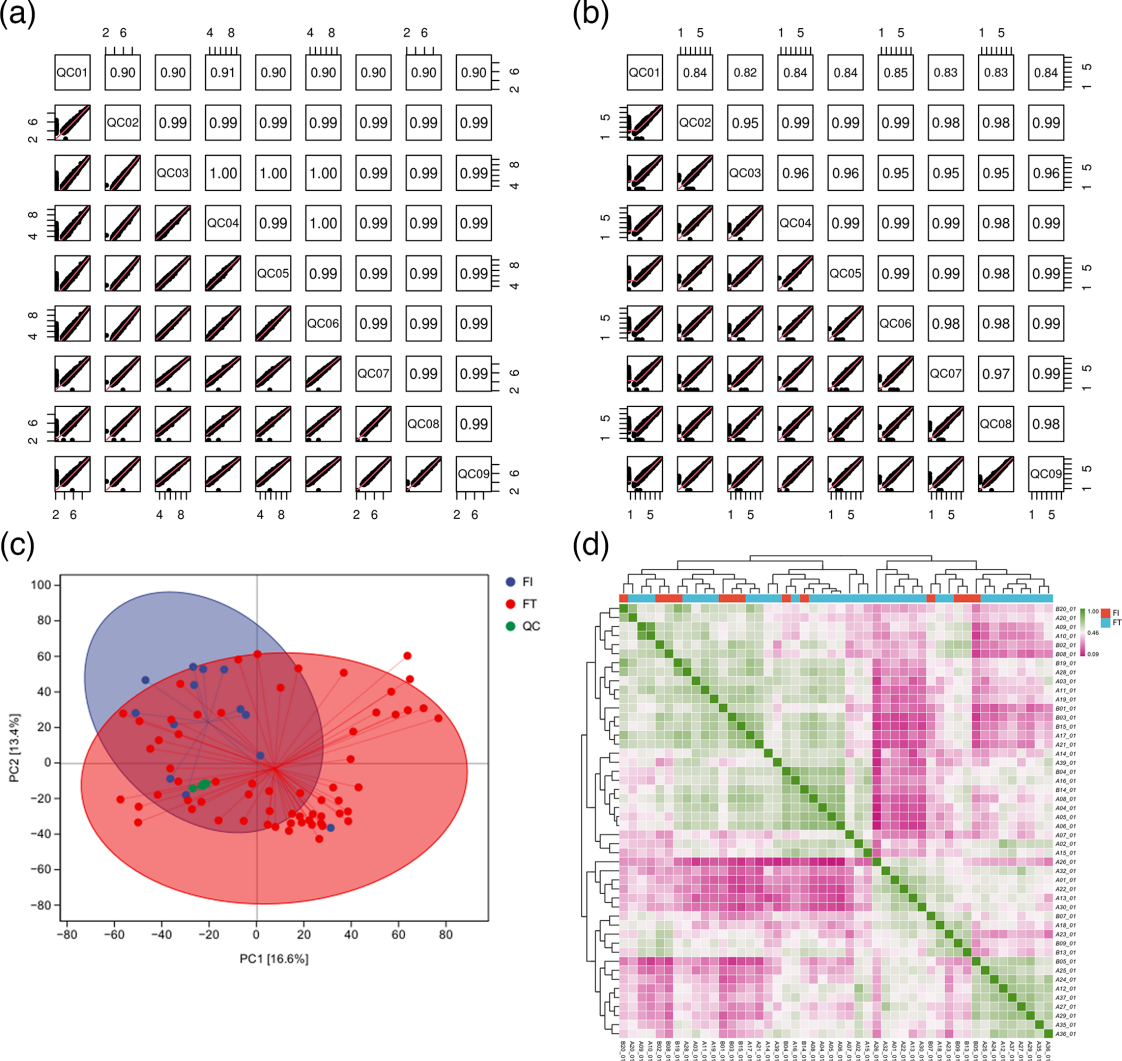
Quality control in metabolomics. (**a**) The QC quality of samples in NEG mode for the two groups. (**b**) The QC quality of samples in POS mode for the two groups. (**c**) The PCA diagrams of the FI and FT groups. (**d**) Analysis of the correlation heatmap for samples in two groups.

In the QC correlation plots, the values represent correlation coefficients. A coefficient greater than 0.9 is considered a very strong correlation. The QC plots in this experiment show that values of each QC sample are close, indicating excellent detection stability, repeatability and reliable data (Fig. 4a, b). In the PCA plot, samples from the FI and FT groups show clear separation in the principal component space, suggesting differences in metabolite composition between the two preterm infant groups. Additionally, the QC samples cluster together, further validating the detection stability (Fig. 4c). The heatmap shows the correlations among samples. The colours represent the correlation strength, with green indicating a strong positive correlation and pink indicating a weak or negative correlation. As can be seen from the figure, the correlations among some samples are high. Moreover, the overall correlation within groups is relatively high, while the correlation among some samples between groups is low, further reflecting the significant differences in the metabolite levels between the FI group and FT group (Fig. 4d).

#### Metabolite identification analysis

**Fig. 5.**
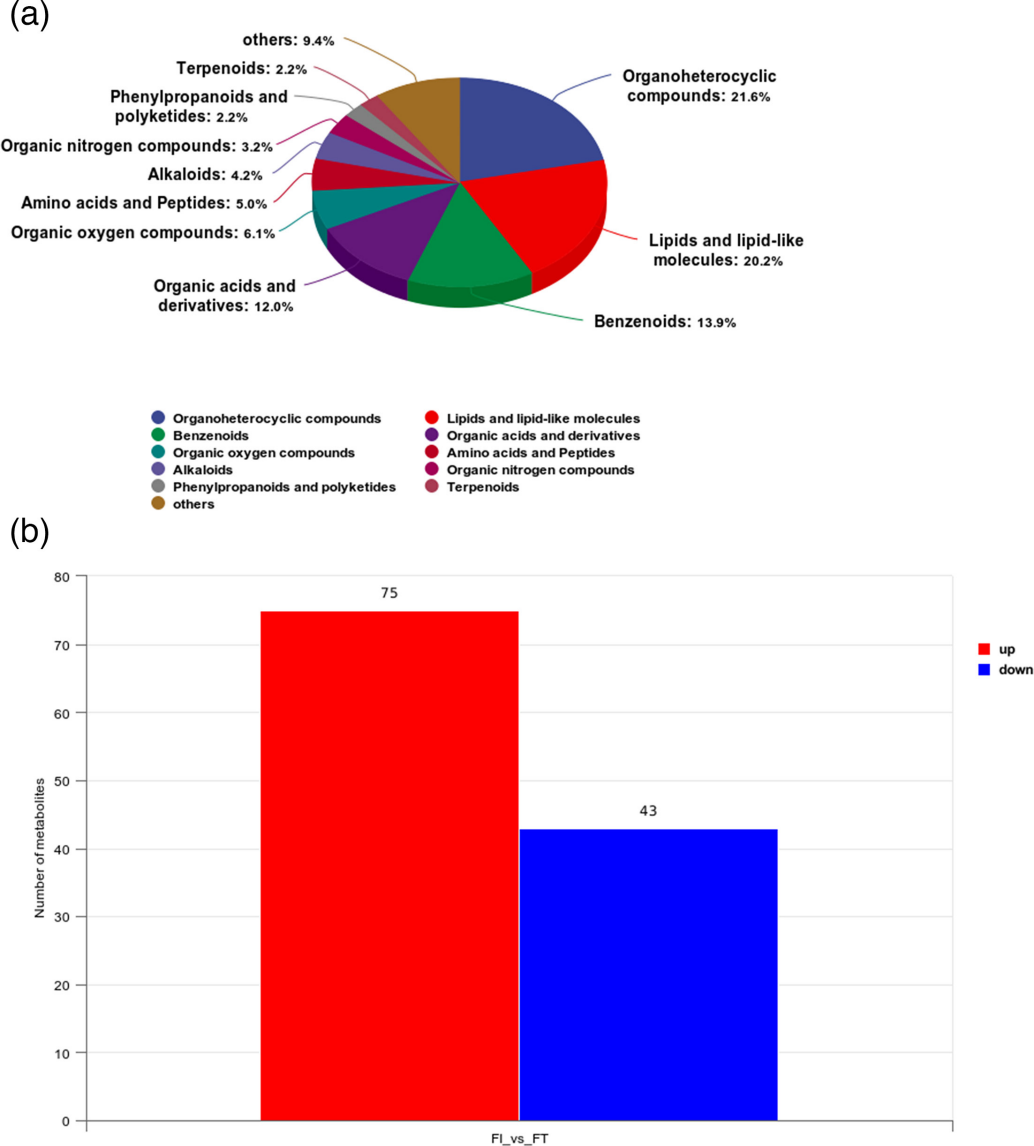
Metabolomic analysis. (**a**) Metabolite identification analysis: different coloured blocks represent different chemical classification entries. The percentage indicates the proportion of the number of metabolites in a chemical classification entry to the total number of identified metabolites. (**b**) Screening of differential substances: the abscissa represents the comparison group, and the ordinate represents the number of differential metabolites.

Identification and analysis of metabolites in the FT and FI groups showed that organic heterocyclic compounds accounted for the highest proportion, reaching 21.6%. Lipids and lipid-like molecules accounted for 20.2%, benzenoids for 13.9% and organic acids and derivatives for 12.0%. Others, such as organic oxygen compounds, alkaloids, amino acids and peptides, are also distributed in varying proportions, but their proportions are relatively small (Fig. 5a).

#### The pairwise comparison and differential analysis between the FT and FI groups

**Fig. 6.**
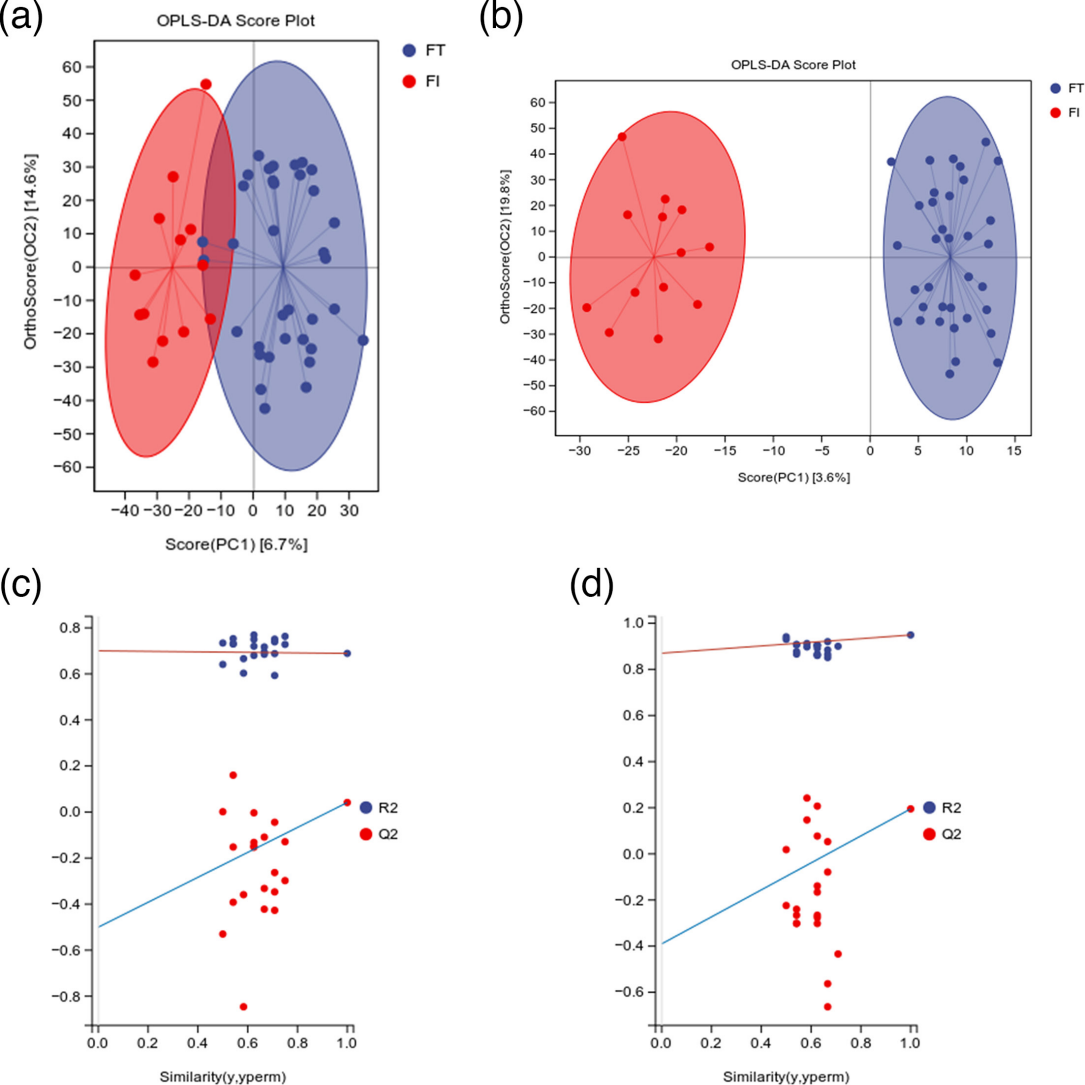
Multivariate statistical analysis of metabolomics. (**a**) Score plot of OPLS-DA analysis in POS ion mode for multivariate statistics. (**b**) Score plot of OPLS-DA analysis in NEG ion mode for multivariate statistics. (**c**) Permutation test plot of OPLS-DA analysis in POS ion mode for multivariate statistics. (**d**) Permutation test plot of OPLS-DA analysis in NEG ion mode for multivariate statistics.

Based on VIP >1 and *P*<0.05, differential metabolites between the two groups were examined. According to the results, the FT group had 43 metabolites that were downregulated and 75 metabolites that were upregulated when compared to the FI group (Fig. 5b). The ropls package in the R language was used for OPLS-DA analysis. Cross-validation and permutation tests were run on the OPLS-DA model to confirm its dependability. From the score plots drawn by OPLS-DA for the analysis of the FT and FI groups, it can be seen that there are clear and distinct clusters between the two groups, and there are significant differences between them (Fig. 6a, b). From the permutation test plots of the two groups, it can be seen that the intersection points of the Q2 regression lines of the two groups with the ordinate are both less than 0. This indicates that the OPLS-DA model established based on the experimental data has no overfitting, and the analysis of differential metabolites is relatively accurate, which can be used for subsequent experimental analysis (Fig. 6c, d).

#### Machine learning analysis of differential substances between two groups

**Fig. 7.**
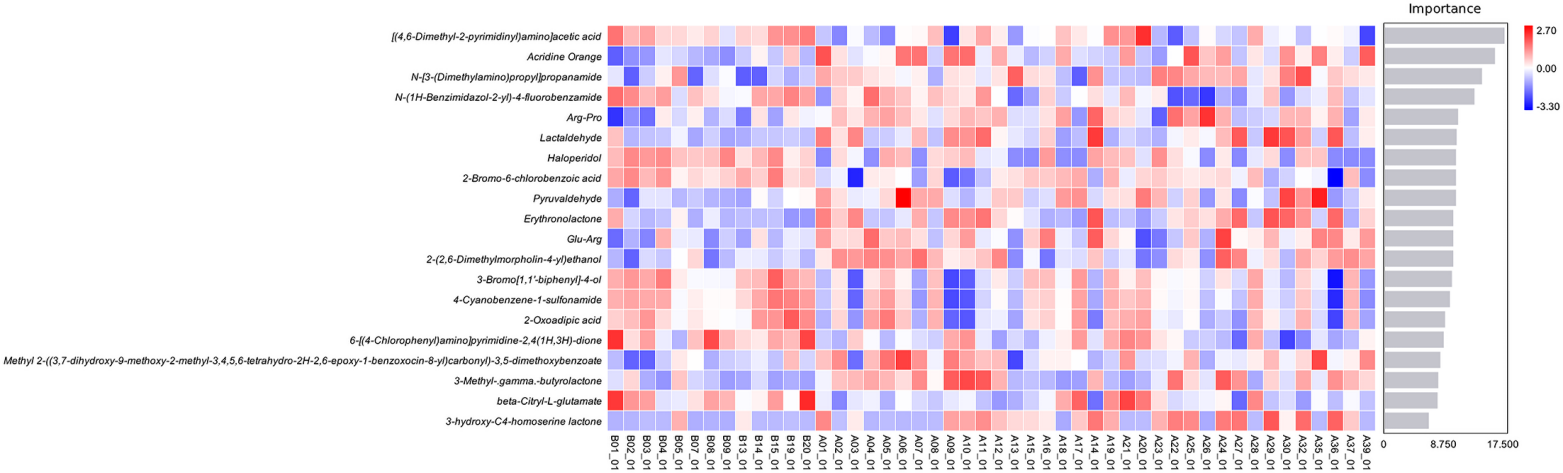
Random forest plot of differential metabolites.

Analysis of the chemicals that differed between the FT and FI groups was done. The *x*-axis of the bar chart displays the species’ relevance scores for the classifier model, while the *y*-axis displays the names of the metabolites. The top 20 metabolites in each sample are displayed in abundance levels on the heatmap (Fig. 7).

#### KEGG functional annotation and enrichment analysis of differential metabolites

**Fig. 8.**
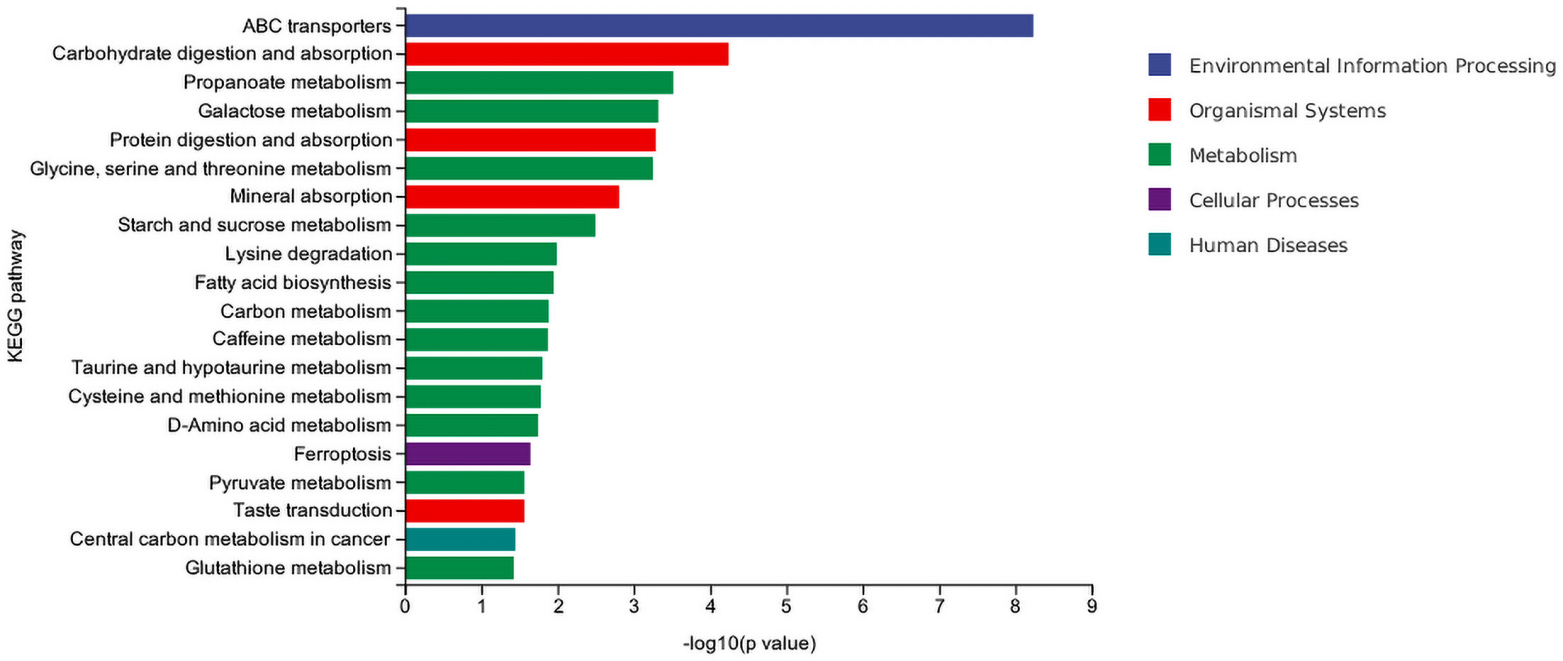
KEGG enrichment analysis of differential metabolites.

KEGG analysis of feeding tolerance and intolerance in preterm infants indicated that multiple metabolic pathways and biological processes differed significantly between the two groups. Among them, the ABC transporters pathway had the highest -log10 (*P*-value) and fell into the category of environmental information processing. Additionally, metabolism-related pathways, such as carbohydrate digestion and absorption and propionate metabolism, as well as organismal systems-related pathways like protein digestion and absorption, also showed significant differences (Fig. 8).

#### Integrated analysis of gut microbiota and metabolites for potential biomarker discovery

**Fig. 9.**
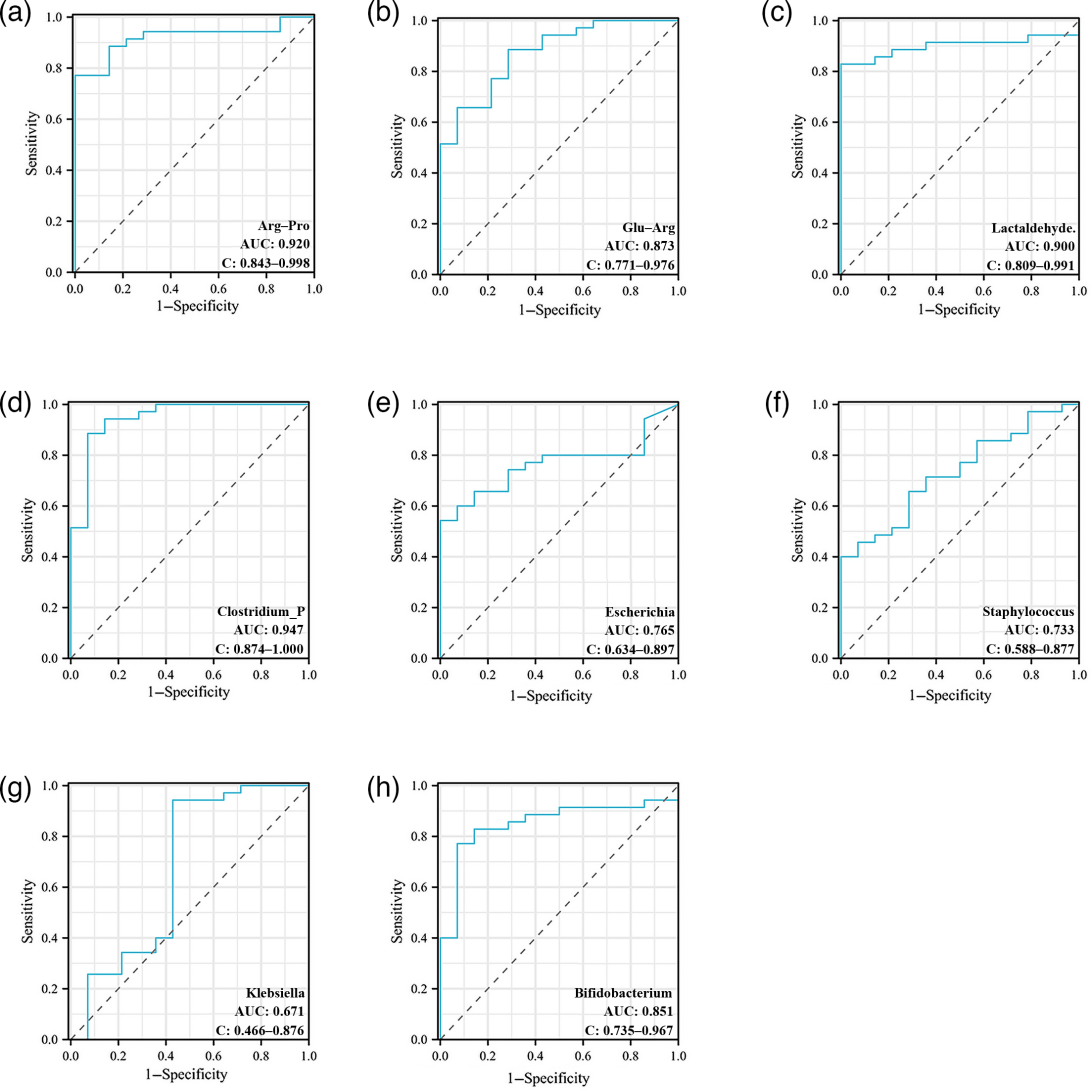
ROC curve. (**a**) Arg–Pro. (**b**) Glu–Arg. (**c**) Lactaldehyde. (**d**) *Clostridium_P*. (**e**) *Escherichia*. (**f**) *Staphylococcus*. (**g**) *Klebsiella*. (**h**) *Bifidobacterium.*

Based on the combined analysis of the target differential microbiota (*P*<0.05) and the differential metabolites, three metabolites – Arg–Pro, Glu–Arg and lactaldehyde – were identified. These metabolites were, respectively, associated with the activities of differential microbiota, including *Bifidobacterium*, *Escherichia*, *Klebsiella*, *Clostridium*_*P* and *Staphylococcus*. Therefore, the ROC curves were drawn for the above gut microbiota and differential metabolites (Fig. 9). The results showed that the AUC values of Arg–Pro, Glu–Arg, lactaldehyde, *Clostridium_P*, *Escherichia*, *Staphylococcus* and *Bifidobacterium* were 0.920, 0.873, 0.900, 0.947, 0.765, 0.733 and 0.851, respectively, indicating that these substances had certain predictive value for evaluating the feeding tolerance of preterm infants. However, the AUC value of *Klebsiella* was 0.671, indicating a limited predictive ability.

## Discussion

FI in preterm infants is a common clinical issue in neonatal intensive care units, which is closely associated with intestinal microecological imbalance and metabolic disorders. Currently, the pathogenesis of feeding intolerance in preterm infants remains incompletely understood, but it is widely believed to be related to gastrointestinal dysfunction such as immature gastrointestinal development, gastrointestinal ischaemia–reperfusion injury and inflammatory responses [15,17]. As a key tool to reveal intestinal metabolic status, faecal metabolomics can reflect the metabolic activity of gut microbiota and the host–microbe interaction by detecting dynamic changes of small-molecule metabolites in faeces [18]. Recent studies have shown that preterm infants are prone to disruptions in metabolic pathways due to immature intestinal development and abnormal microbial colonization, which affect nutrient absorption and intestinal barrier function [19]. Therefore, understanding the characteristics of gut microbiota and faecal metabolome in low-birth-weight preterm infants is helpful for early detection and intervention of feeding intolerance, the improvement of intestinal health and the provision of objective evidence for the diagnosis of FI.

The data of this study showed that the incidence of clinical symptoms, including vomiting, abdominal distension and gastric residue (>30% or >2 ml kg^−1^), was significantly higher in preterm infants of the FI group than in those of the FT group. This difference was observed at the 21-day-old evaluation (*P*<0.001). Analysis of gut microbiota at the genus level revealed significant differences between the FI group and FT group: pro-inflammatory genera such as *Clostridium_P* (3.93%) and *Burkholderia* (4.06%) were abnormally enriched in the FI group, while these genera were not detected in the FT group. Conversely, the proportions of *Escherichia coli* (10.92%), *Bifidobacterium* (3.16%) and *Klebsiella* (6.88%) were significantly increased in the FT group. This suggests that changes in the gut microbiota of preterm infants are associated with the occurrence of feeding intolerance. The gut microbiota and the human body have a symbiotic relationship; they help the body maintain intestinal barrier function and contribute to the host’s nutrition. If the homeostasis of the gut microbiota is disrupted, its resistance to the colonization of pathogenic bacteria decreases, thereby easily triggering related diseases such as inflammatory bowel disease [20]. The establishment of gut microbiota in neonates is a slow and dynamic process, and preterm infants are one of the special groups among neonates, making them more likely to affect the colonization ability and timing of gut microbiota in the body, which in turn can influence the species diversity of intestinal micro-organisms [21]. The results of this study showed that the proportion of the genus *Bifidobacterium* in the gut microbiota of the FT group was higher than that in the FI group. As a key probiotic in the intestine of preterm infants, *Bifidobacterium* can promote the production of SCFAs, enhance intestinal barrier function and inhibit pathogen colonization [22]. Consistent with the findings of Yuan *et al.* [23], the abundances of beneficial bacteria such as *Bifidobacterium* and *Lactobacillus* were significantly reduced in the gut microbiota of preterm infants with FI. This may be related to the development of tolerance in preterm infants.

Specifically, the significant enrichment of *Clostridium_P* in the FI group may be associated with intestinal dysbiosis, which is one of the important predisposing factors for NEC in preterm infants [24,25]. As demonstrated by Pammi *et al.* [26], certain species of *Clostridium* are significantly increased in NEC patients, suggesting their potential role in intestinal inflammatory responses. Additionally, the enrichment of *Burkholderia* and *Limosilactobacillus* in the FI group is also associated with intestinal dysbiosis, which may further exacerbate intestinal barrier dysfunction and increase the risks of FI and NEC [26]. In contrast, the FT group is characterized by dominant colonization of *Escherichia*, *Klebsiella* and *Bifidobacterium*, which aligns with the features of protective intestinal microbiota. Although *Escherichia* and *Klebsiella* may be considered as conditional pathogens in some contexts, they have also shown protective effects in certain studies, particularly in maintaining intestinal microbial balance. *Bifidobacterium*, as a probiotic, its dominant colonization in the intestine is typically closely associated with enhanced intestinal health and immunomodulatory functions [27].

The metabolomics KEGG enrichment analysis showed significant enrichment of the ABC transporter pathway in both groups. Among ABC transporters, ABCB1/P-glycoprotein plays a key role in maintaining intestinal homeostasis by actively effluxing bacterial metabolites and toxins. In animal models lacking ABCB1, the incidence of gut microbiota dysbiosis and ulcerative colitis increased significantly [28]. The downregulation of the propionate metabolism pathway reflects the insufficient production of SCFAs, which is consistent with the reduced abundance of SCFA-producing genera (such as *Bifidobacterium* and *Veillonella_A*) in the FI group. The abnormality of the propionate metabolism pathway in this study may be due to the colonization disorder of acid-producing bacteria, such as *Limosilactobacillus.* This reduces propionic acid production, weakens energy supply to intestinal epithelial cells and worsens feeding intolerance symptoms. In addition, the disorder of the carbohydrate digestion and absorption pathway may be related to lactose malabsorption caused by the abnormal colonization of *Limosilactobacillus* and other genera, which is consistent with the common manifestation of lactose intolerance in FI children [29]. This study also found that microbial-related metabolites such as Arg-Pro (AUC=0.920) and Glu-Arg (AUC=0.873) had excellent predictive value for FI. Together, these findings support a new pathological hypothesis: FI may result from a reduction in protective bacteria (such as *Bifidobacterium*), causing a deficiency in beneficial metabolites, including SCFAs and Arg-Pro. Concurrently, overgrowth of conditional pathogens (such as *Clostridium_P*) is producing toxic metabolites, which disrupt the enteric nervous system function.

In terms of the diagnostic value of SCFAs, although this study did not directly measure SCFA concentrations, abnormalities in related metabolic pathways (e.g. propanoate metabolism) and changes in the abundance of SCFA-producing genera (e.g. *Bifidobacterium* and *Clostridium_P*) suggest that SCFAs may serve as potential diagnostic markers. Specifically, *Bifidobacterium*, a key propionate-producing genus, showed reduced abundance in the FI group, which may indicate a decrease in propionate levels. Propionate has been confirmed to regulate intestinal immunity through the GPR43 receptor, and its deficiency may enhance intestinal inflammatory responses [30]. Additionally, *Clostridium_P*, a candidate butyrate-producing genus, showed significantly increased abundance in the FI group, which may indicate disrupted microbial metabolic functions rather than increased acid production. In this study, both *Bifidobacterium* (AUC=0.851) and *Clostridium_P* (AUC=0.947) could serve as diagnostic criteria for feeding tolerance in preterm infants.

## Conclusion

Our study demonstrates that certain bacterial genera in the gut microbiota, such as *Staphylococcus*, *Clostridium*_*P*, *Bifidobacterium* and *Escherichia*, can serve as diagnostic criteria for feeding tolerance in preterm infants. Additionally, metabolites identified in metabolomics, including Arg–Pro, Glu–Arg and lactaldehyde, also serve this role. *Klebsiella* shows a certain degree of diagnostic efficacy but falls into the category of ‘low accuracy’, requiring comprehensive evaluation considering the research background, sample characteristics and clinical context.

## Supplementary material

10.1099/jmm.0.002138 Uncited Table S1.

## Abbreviations

AUC: area under the curve
EMR: electronic medical record
FI: feeding intolerance
FT: feeding-tolerant
GRV: gastric residual volume
NCBI: National Center for Biotechnology Information
NEC: necrotizing enterocolitis
OPLS-DA: orthogonal projections to latent structures discriminant analysis
PCA: principal component analysis
QC: quality control
ROC: receiver operating characteristic
SCFAs: short-chain fatty acids
